# An Investigation of the Relationship Between Carotid Artery Stenosis and White Matter Hyperintensities

**DOI:** 10.7759/cureus.39468

**Published:** 2023-05-25

**Authors:** Sedat Yasin, Rabia Tasdemir

**Affiliations:** 1 Department of Neurology, Faculty of Medicine, Gaziantep University, Gaziantep, TUR; 2 Department of Anatomy, School of Medicine, Gaziantep Islam Science and Technology University, Gaziantep, TUR

**Keywords:** volume, white matter lesion, magnetic resonance imaging, stenosis, internal carotid artery

## Abstract

Introduction

White matter hyperintensities (WMHs) are frequently detected on magnetic resonance imaging (MRI) with age. Although the etiology of WMH has not been fully explained, it was reported to be associated with internal carotid artery (ICA) stenosis as well as small vessel diseases. The number and volume of these lesions might increase in cases of internal carotid artery (ICA) stenosis.

The present study aimed to calculate the localization and volumes of white matter lesions in the VolBrain Program and investigate the relationship between age and sex in patients with symptomatic and asymptomatic internal carotid artery stenosis.

Methods

MRI scans of patients with carotid stenosis with T1-weighted and fluid-attenuated inversion recovery (FLAIR) sequences were performed retrospectively for the present study, which had a retrospective design. The patients were divided into two groups (<70% and ≥70%) because endovascular intervention was considered in patients with asymptomatic stenosis over 70%. Digital subtraction angiography was used to detect carotid artery stenosis. They were also divided into four groups. According to laterality and degree of stenosis, ICA stenosis was separated as those with <70% stenosis on both sides (group 1), right side ICA <70%, left side ≥70% stenosis (group 2), right side ICA ≥70%, left side <70% stenosis (group 3), and ≥70% stenosis on both sides (group 4). A total of 102 patient images were selected that met the inclusion criteria. The measurements of white matter lesion volumes were made using the LesionBrain application in the VolBrain Program.

Results

The MRI of 82 patients (mean age: 65.55 ± 9.28 years), 28 females and 54 males, were used in the present study. According to LesionBrain Analysis, the total WMH volume was seen in the first and third groups at most. When analyzed in regional terms, stenosis was mostly detected in the first and third groups in the periventricular region. WMH volume was less in all areas in group 4. When examined according to the number of lesions, the most lesions were detected in the third group in the juxtacortical region. When the difference between the groups was examined, a significant difference was detected only in the volume change in the deep white region (p=0.011). No significant differences were found between WMH volumes and age and gender (p>0.05).

Conclusion

Stenosis of the external internal carotid artery might cause hypoperfusion and silent embolization in the brain. As a result, as well as pathological conditions in cortical areas, ischemic areas in the white matter might also cause cognitive disorders.

## Introduction

Atherosclerosis in the internal carotid artery (ICA) might cause ischemic strokes [1]. ICA occlusions because of atherosclerosis account for 4%-15% of all ischemic stroke incidence [2-4]. It is also suggested that ICA stenosis causes loss of cognitive functions [1]. Previous studies reported that high-grade stenosis of the ICA, through hypoperfusion and silent embolization, might be an independent risk factor for cognitive disorders and even dementia [5].

White matter hyperintensities (WMHs) are cerebral lesions considered to have a vascular origin and appear incidentally on magnetic resonance imaging (MRI) [1,6]. Although the pathogenesis of WMH is not fully understood, it might have multifactorial causes such as hypertension, diabetes, hyperlipidemia, smoking, and dementia [5,7-10]. There are also studies reporting that WMH is associated with cognitive impairment and neurological disorders, which increase the risk of stroke, dementia, and depression [6,11]. Although the etiology of WMH is not fully explained, it was reported to be associated with ICA stenosis as well as small vessel diseases [6,12].

Although endovascular interventions are considered by interventional neurologists in patients with asymptomatic ICA stenosis ≥70%, WMH might be seen in patients with <70% asymptomatic ICA stenosis, although endovascular intervention is not considered [2,4,13]. It is not clear whether there is a relationship between the severity of ICA stenosis and the volume of WMH. Determining the relationship between ICA stenosis and the localization and volume of WMH is important for the development of strategies to prevent WMH and its associated clinical results. Although there are literature data reporting the relationship between ICA stenosis and WMH, it was mostly studied in patients with stroke and intracranial vascular disease [14,15]. The present study aimed to investigate whether there is a relationship between the degree of stenosis and the volume of WMH in patients with external ICA stenosis and no other pathological findings.

## Materials and methods

The study was conducted on the MRI of people with ICA stenosis who applied to the Neurology Clinic of Gaziantep University Faculty of Medicine between 2017 and 2022. The presence and degree of ICA stenosis were determined by a specialist neurologist.

Inclusion criteria

The inclusion criteria were as follows: a diagnosis of ICA stenosis with digital subtraction angiography (DSA) procedure and the presence of fluid-attenuated inversion recovery (FLAIR) sequence on MRI.

Exclusion criteria

The exclusion criteria were as follows: a diagnosis of hypertension, diabetes, and other neurological or psychiatric diseases, previous ischemic stroke, or endovascular procedure.

Approximately 3,000 patients were analyzed retrospectively for the study. The MRI of 102 patients who were diagnosed with ICA stenosis and met the exclusion criteria from ~2,000 patients who had MRI was used. The calculations could be made on the MRI of 82 patients (age range: 37-92 years) in the LesionBrain Program, which was used to calculate the volume. ICA stenosis was classified as those with stenosis below 70% and those with 70% and above, which were determined as the criteria for endovascular intervention in asymptomatic individuals.

MRI protocol

**Figure 1:**
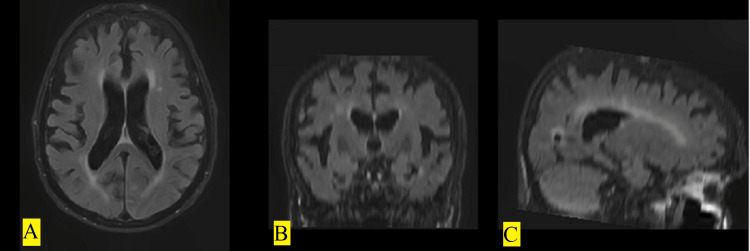
Magnetic resonance images in FLAIR sequence in (A) transverse, (B) coronal, and (C) sagittal planes. FLAIR: fluid-attenuated inversion recovery

Gyroscan Intera VCT XTe Light speed#3500/1.5 Tesla MRI devices (Philips, Amsterdam, Netherlands) were used for the MRI of the patients. The parameters used were as follows: repetition time (TR), 11 ms; echo time (TE), 5 ms; inversion time (TI), -; section thickness, 1 mm; and section void, 0 mm in the protocol of 3D T1-weighted images (3DT1) from the MR series and TR, 11,000 ms; TE, 140 ms; TI, 2,500 ms; section thickness, 5 mm; and section void, 0.5 mm in the protocol of FLAIR images (Figure 1).

**Figure 2:**
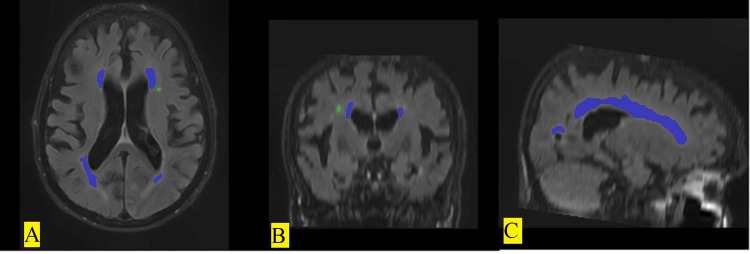
Regions with WMH as a result of LesionBrain segmentation in the (A) transverse, (B) coronal, and (C) sagittal planes are shown in color. WMH: white matter hyperintensity

Segmentation and volume calculation was performed with the LesionBrain, which is an online volume calculation tool, on the obtained MRI through T1 and FLAIR Sequences. Radiological images in Digital Imaging and Communications in Medicine (DICOM) format were converted into “.nifti” extension with the open source program MRIcron (www.nitrc.org). The images that were converted into the appropriate format were uploaded to the VolBrain (https://www.volbrain.upv.es/) 59 automated segmentation module for preprocessing and volume measurements (Figure 2). With proven reliability [16], the VolBrain segments the brain structures and reports the location of WMHs in numbers, volume measurements in cm^3^, and percent.

Statistical analysis

All statistical analyzes were made using the Statistical Package for the Social Sciences (SPSS) for Windows version 22.0 (IBM SPSS Statistics, Armonk, NY, USA). The conformity of the data to the normal distribution was checked. When it was determined that it was not suitable for the normal distribution, the Kruskal-Wallis test, which is one of the nonparametric tests, was used for the comparisons between the groups. The Spearman correlation analysis was used to determine the relationship between the variables. The relationship between categorical variables was analyzed using the chi-square analysis. The statistical significance level was taken as p<0.05.

## Results

The study was conducted on the brain MRI of 82 patients (mean age: 65.55 ± 9.28 years) (28 females and 54 males). According to laterality and degree of stenosis, ICA stenosis was separated as those with <70% stenosis on both sides (group 1), right side ICA <70%, left side ≥70% stenosis (group 2), right side ICA ≥70%, left side <70% stenosis (group 3), and ≥70% stenosis on both sides (group 4). There were 17 patients in group 1 (seven females and 10 males), 21 patients in group 2 (five females and 16 males), 28 patients in group 3 (nine females and 19 males), and 16 patients in group 4 (seven females and nine males) (Table 1).

**Table 1:** Distribution of groups formed according to the laterality and degree of stenosis by gender.

Sex	Group 1	Group 2	Group 3	Group 4	Total
Female	7	5	9	7	28
Male	10	16	19	9	54
Total	17	21	28	16	82

According to the LesionBrain Analysis, the volumes of WMHs were calculated separately in total, periventricular, juxtacortical, and deep white regions. In this regard, the total WMH volume was mostly detected in the first and third groups. When analyzed regionally, it was mostly detected in the first and third groups in the periventricular region. Interestingly, patients with ≥70% stenosis on both sides had less WMH volume in all areas. The mean and standard deviation of the regional volumes according to the degree of stenosis are given in Table 2.

**Table 2:** Distribution of the volumes of regions with WMH according to stenosis groups. WMH: white matter hyperintensity

Regions with WMH	Stenosis group	Mean (cm^3^) ± standard deviation
Periventricular region volume	Group 1	17.34 ± 20.88
	Group 2	13.38 ± 24.35
	Group 3	14.61 ± 22.77
	Group 4	5.59 ± 10.73
Juxtacortical region volume	Group 1	0.53 ± 0.50
	Group 2	1.19 ± 1.78
	Group 3	2.74 ± 6.29
	Group 4	0.47 ± 0.48
Deep white region volume	Group 1	0.07 ± 0.08
	Group 2	0.14 ± 0.17
	Group 3	0.21 ± 0.35
	Group 4	0.10 ± 0.19
Total WMH volume	Group 1	17.94 ± 20.93
	Group 2	14.72 ± 24.09
	Group 3	17.55 ± 22.26
	Group 4	6.16 ± 11.1

When examined according to the number of lesions, the most lesions were detected in group 3 in the juxtacortical region. Although the least lesions were detected in group 1 in the juxtacortical and periventricular regions, the least lesions were observed in group 4 in the deep white region. The distribution of lesion numbers and percentages according to regions and stenosis groups is given in Table 3.

**Table 3:** Distribution of the number and percentage of WMHs by region and stenosis groups. N: number, %: percentage, WMH: white matter hyperintensity

	Periventricular lesion count (N (%))	Juxtacortical lesion count (N (%))	Deep white lesion count (N (%))	Total lesion count (N (%))
Group 1	82 (0.22%)	118 (0.20%)	80 (0.21%)	280 (0.21%)
Group 2	90 (0.24%)	165 (0.27%)	110 (0.29%)	365 (0.27%)
Group 3	113 (0.30%)	190 (0.31%)	134 (0.35%)	438 (0.32%)
Group 4	90 (0.24%)	186 (0.31%)	54 (0.14%)	276 (0.20%)
Total	375	605	378	1,359

When the difference between the groups was examined, significant differences were detected only in the volume change in the deep white region (p=0.011). When the deep white region was examined, it was found that the difference was between group 4 and groups 2 and 3 (p<0.05) (Table 4).

**Table 4:** Distribution of regions with WMH according to stenosis groups. a,b: different letters indicate difference between the types WMH: white matter hyperintensity

	Stenosis group				p
	Group 1	Group 2	Group 3	Group 4	
	Median (Q1-Q3)	Median (Q1-Q3)	Median (Q1-Q3)	Median (Q1-Q3)	
Periventricular region	7.85 (1.98-31.52)	2.20 (0.29-11.77 )	7.62 (0.34-16.24 )	1.90 (0.19-6.74)	0.208
Juxtacortical region	0.49 (0.12-0.71)	0.39 (0.07-1.56)	0.25 (0.07-1.03)	0.34 (0.16-0.57)	0.967
Deep white region	0.04 (0.01-0.11)	0.12 (0.05-0.21)^a^	0.09 (0.04-0.22)^a^	0.005 (0-0.14)^b^	0.011

When the relationship of WMH volumes grouped according to stenosis laterality and degrees with gender was examined, no significant relationships were detected between the groups and gender (p>0.05). We could not analyze the degree of stenosis and WMH volumes by age group because there was only one patient under the age of 45 and not all groups had enough patients for analysis. No correlation was detected between WMH volumes and age (p=0.2, r=-0.13). Considering the relationship between the variables, a low-intensity (p=0.005, r=0.31) and positive correlation was detected between WMH volumes in the deep white region and WMH volumes in the juxtacortical region (Table 5).

**Table 5:** Relationship of regions with WMH with each other and with age. *0.2<r<0.4 (low correlation) **0.4<r<0.6 (medium correlation) r: correlation coefficient, N: number, WMH: white matter hyperintensity

Spearman correlation		Total volume (cm^3^)	Periventricular volume (cm^3^)	Juxtacortical volume (cm^3^)	Deep white volume (cm^3^)
Age	r	-0.134	-0.070	-0.154	-0.153
	p	0.232	0.532	0.167	0.169
	N	82	82	82	82
Total volume (cm^3^)	r		0.850^**^	0.396^**^	0.250^*^
	p		0.000	0.000	0.024
	N		82	82	82
Periventricular volume (cm^3^)	r			0.121	0.176
	p			0.279	0.114
	N			82	82
Juxtacortical volume (cm^3^)	r				0.310^**^
	p				0.005
	N				82

## Discussion

WMHs cause a pathophysiological condition that affects cognitive, mental, and physical function negatively with age [9]. In addition to cerebrovascular diseases, WMHs are also associated with cardiovascular diseases, diabetes, hypertension, and neurological and psychiatric disorders [6,8,9, 11,17-20]. Although there are so many different causes of WMH, all criteria were excluded, and the purpose was to investigate only its relationship with ICA stenosis. For this reason, the sample was small.

Although many studies are reporting that WMH increases with age [7,9,18,21-24], in a previous study that compared those with extracranial and intracranial atherosclerotic stenosis, no relationship was found between WMH and age in those with extracranial atherosclerotic stenosis [15]. In the present study, to support the study of Park et al. [15], no relationship was detected between WMH and age. The reason why our findings did not overlap with the studies that found a relationship between age and WMH might be because of the small number of our sample, as well as the fact that diseases such as hypertension, diabetes, and dementia, which increase with age, were excluded.

It was reported in a prospective study that according to Doppler ultrasound, people with 50%-70% carotid stenosis had a higher number and volume of new WMHs than those with stenosis below 50%, although women with moderate carotid stenosis had a higher number and volume of WMH [25]. In the present study, it was determined that the total WMH volume was higher in patients with bilateral stenosis <70% (group 1) and right-sided stenosis grade ≥70% and in patients with left-sided stenosis <70% (group 3), and the number of lesions was higher in those with unilateral carotid stenosis ≥70% (Tables 2, 3). However, no significant differences were detected between the groups. In the study of Ammirati et al. [25], the absence of any restrictions other than cardiovascular diseases and the stricter restriction in our study might cause the results to be inconsistent. Potter et al. [14], on the other hand, did not find any relationships between WMH and ipsilateral carotid stenosis in their study conducted on patients with ischemic stroke, which supports our study. In another study, Altaf et al. [24] could not find any relationships between WMH volume and carotid stenosis when they did not consider the age parameter. Likewise, in a prospective study examining patients with low-grade carotid stenosis, they stated that the degree of carotid stenosis was not effective on WMH volume at the end of 12 years [26]. Baradaran et al. [27] reported that infarcts increased in cortical areas in >50% of people with ICA stenosis, but there was no significant difference in WMH.

In their study examining the relationship between carotid stenosis and WMH, Kandiah et al. [10] reported that WMH volume in the periventricular region increased with the degree of stenosis, and although this was statistically significant, they could not find a significant difference between deep subcortical region WMH volumes and the degree of stenosis. In our study, contrary to the study of Kandiah et al., no significant differences were detected in volumes varying with the degree of stenosis in the periventricular region. The researchers observed that only WMH volumes in the deep white region changed negatively with the degree of stenosis and were statistically significant. We think that this difference occurred because Kandiah et al. worked on patients with acute lacunar infarction, although those without clinical findings were included in our study. In their study, which they conducted without any restrictions in terms of diabetes, hypertension, and cardiovascular diseases, Saba et al. [28] reported that they found the WMH volume in the periventricular region to be significantly higher than the WMH volume in the deep white region. In the present study, although the most WMH was detected in the periventricular region in terms of volume, there were more WMHs in the juxtacortical area in number. Also, when the volumetric relationship was evaluated, it was found that there was a low-intensity positive correlation between the WMH volume in the juxtacortical area and the WMH volume in the deep white area. When the findings were compared in terms of volume, our findings coincided with the study of Saba et al. However, since the juxtacortical area was not specified in the study of Saba et al., a comparison cannot be made in terms of WMH numbers.

Limitations

One of the main limitations of the present study was the small number of subjects, which would reduce the statistical significance. The strict exclusion criteria in the study both reduced the number of subjects and limited the factors to compare. Another limitation was that patient information was obtained from the hospital registry system because the study had a retrospective nature. The study included those without any diabetes, hypertension, cerebrovascular and cardiovascular diseases, and neurological or psychiatric disease diagnosis and/or treatment; if one of these unrecorded diseases was present, it might affect the results. Another limitation is that because the study was retrospective, a cognitive assessment could not be performed on the patients.

## Conclusions

The volume and number of WMH were not associated with age or gender in patients with stenosis of the extracranial internal carotid artery who were not diagnosed with a disease that would affect WMH. When the researchers examined the WMH volumes according to regions, they were detected more in the periventricular region in volume and the juxtacortical region in number. Considering the relationship between the regions where WMH volumes were seen, a low-intensity positive correlation was detected between the juxtacortical region and the deep white region. The total WMH volume correlated positively with the periventricular region at a high intensity, at a moderate level with the juxtacortical region, and at a low intensity with the deep white region.

In conclusion, stenosis in the external internal carotid artery might cause hypoperfusion and silent embolization in the brain. As a result, although cognitive evaluation could not be performed in this study, it should be considered that pathological conditions in cortical regions as well as ischemic areas in white matter may cause cognitive disorders.

## References

[1] Porcu M, Sanfilippo R, Montisci R, Balestrieri A, Suri JS, Wintermark M, Saba L (2020). White-matter hyperintensities in patients with carotid artery stenosis: an exploratory connectometry study. Neuroradiol J.

[2] Bonati LH, Jansen O, de Borst GJ, Brown MM (2022). Management of atherosclerotic extracranial carotid artery stenosis. Lancet Neurol.

[3] Deniz C, Altunan B, Aykaç Ö, Özdemir AÖ (2022). Coexistence of external carotid artery embolus and internal carotid artery occlusion in acute ischemic stroke: an indicator of cardioembolic etiology?. J Stroke Cerebrovasc Dis.

[4] Krawisz AK, Carroll BJ, Secemsky EA (2021). Risk stratification and management of extracranial carotid artery disease. Cardiol Clin.

[5] Dutra AP (2012). Cognitive function and carotid stenosis: review of the literature. Dement Neuropsychol.

[6] Ye H, Wang Y, Qiu J, Wu Q, Xu M, Wang J (2018). White matter hyperintensities and their subtypes in patients with carotid artery stenosis: a systematic review and meta-analysis. BMJ Open.

[7] Guevarra AC, Ng SC, Saffari SE, Wong BY, Chander RJ, Ng KP, Kandiah N (2020). Age moderates associations of hypertension, white matter hyperintensities, and cognition. J Alzheimers Dis.

[8] Mankovsky B, Zherdova N, van den Berg E, Biessels GJ, de Bresser J (2018). Cognitive functioning and structural brain abnormalities in people with type 2 diabetes mellitus. Diabet Med.

[9] Zhuang FJ, Chen Y, He WB, Cai ZY (2018). Prevalence of white matter hyperintensities increases with age. Neural Regen Res.

[10] Kandiah N, Goh O, Mak E, Marmin M, Ng A (2014). Carotid stenosis: a risk factor for cerebral white-matter disease. J Stroke Cerebrovasc Dis.

[11] O'Brien JT (2014). Clinical significance of white matter changes. Am J Geriatr Psychiatry.

[12] Enzinger C, Ropele S, Gattringer T, Langkammer C, Schmidt R, Fazekas F (2010). High-grade internal carotid artery stenosis and chronic brain damage: a volumetric magnetic resonance imaging study. Cerebrovasc Dis.

[13] Kamel H, Gialdini G, Baradaran H (2017). Cryptogenic stroke and nonstenosing intracranial calcified atherosclerosis. J Stroke Cerebrovasc Dis.

[14] Potter GM, Doubal FN, Jackson CA, Sudlow CL, Dennis MS, Wardlaw JM (2012). Lack of association of white matter lesions with ipsilateral carotid artery stenosis. Cerebrovasc Dis.

[15] Park JH, Kwon HM, Lee J, Kim DS, Ovbiagele B (2015). Association of intracranial atherosclerotic stenosis with severity of white matter hyperintensities. Eur J Neurol.

[16] Harkey T, Baker D, Hagen J, Scott H, Palys V (2022). Practical methods for segmentation and calculation of brain volume and intracranial volume: a guide and comparison. Quant Imaging Med Surg.

[17] Meurs M, Roest AM, Groenewold NA (2016). Gray matter volume and white matter lesions in chronic kidney disease: exploring the association with depressive symptoms. Gen Hosp Psychiatry.

[18] Mok V, Wong KK, Xiong Y (2011). Cortical and frontal atrophy are associated with cognitive impairment in age-related confluent white-matter lesion. J Neurol Neurosurg Psychiatry.

[19] Murray A, McNeil C, Salarirad S, Deary I, Phillips L, Whalley L, Staff R (2016). Brain hyperintensity location determines outcome in the triad of impaired cognition, physical health and depressive symptoms: a cohort study in late life. Arch Gerontol Geriatr.

[20] Pu Y, Liu L, Zou X (2009). Relationship between leukoaraiosis and cerebral large artery stenosis. Neurol Res.

[21] Henry Feugeas MC, De Marco G, Peretti II, Godon-Hardy S, Fredy D, Claeys ES (2005). Age-related cerebral white matter changes and pulse-wave encephalopathy: observations with three-dimensional MRI. Magn Reson Imaging.

[22] Honda Y, Noguchi A, Maruyama K (2015). Volumetric analyses of cerebral white matter hyperintensity lesions on magnetic resonance imaging in a Japanese population undergoing medical check-up. Geriatr Gerontol Int.

[23] Poggesi A, Pracucci G, Chabriat H (2008). Urinary complaints in nondisabled elderly people with age-related white matter changes: the Leukoaraiosis And DISability (LADIS) Study. J Am Geriatr Soc.

[24] Altaf N, Morgan PS, Moody A, MacSweeney ST, Gladman JR, Auer DP (2008). Brain white matter hyperintensities are associated with carotid intraplaque hemorrhage. Radiology.

[25] Ammirati E, Moroni F, Magnoni M (2019). Progression of brain white matter hyperintensities in asymptomatic patients with carotid atherosclerotic plaques and no indication for revascularization. Atherosclerosis.

[26] Ghaznawi R, Vonk JM, Zwartbol MH, Bresser J, Rissanen I, Hendrikse J, Geerlings MI (2023). Low-grade carotid artery stenosis is associated with progression of brain atrophy and cognitive decline. The SMART-MR study. J Cereb Blood Flow Metab.

[27] Baradaran H, Gialdini G, Mtui E, Askin G, Kamel H, Gupta A (2016). Silent brain infarction in patients with asymptomatic carotid artery atherosclerotic disease. Stroke.

[28] Saba L, Lucatelli P, Anzidei M, di Martino M, Suri JS, Montisci R (2018). Volumetric distribution of the white matter hyper-intensities in subject with mild to severe carotid artery stenosis: does the side play a role?. J Stroke Cerebrovasc Dis.

